# Advancing nitrogen nutrition index estimation in summer maize using continuous wavelet transform

**DOI:** 10.3389/fpls.2024.1478162

**Published:** 2024-11-11

**Authors:** Mingxia Wang, Ben Zhao, Nan Jiang, Huan Li, Jiumao Cai

**Affiliations:** ^1^ School of Hydraulic Engineering, Yellow River Conservancy Technical Institute, Kaifeng, China; ^2^ College of Tobacco Science, Henan Agricultural University, Zhengzhou, China; ^3^ Farmland Irrigation Research Institute, Chinese Academy of Agricultural Sciences, Xinxiang, Henan, China

**Keywords:** maize, critical nitrogen concentration, nitrogen nutrition index, wavelet feature, Mexican Hat

## Abstract

Rapid and non-destructive diagnosis of plant nitrogen (N) status is crucial to optimize N management during the growth of summer maize. This study aimed to evaluate the effectiveness of continuous wavelet analysis (CWA) in estimating the nitrogen nutrition index (NNI), to determine the most suitable wavelet analysis method, and to identify the most sensitive wavelet features across the visible to near-infrared spectrum (325–1,025 nm) for accurate NNI estimation. Field experiments were conducted across two sites (Kaifeng and Weishi) during the 2022 and 2023 growing seasons using four summer maize cultivars (XD20, ZD958, DH661, and DH605) under varying N application rates (0, 80, 160, 240, and 320 kg N ha^-1^). Canopy reflectance spectra and plant samples were collected from the V6 to V12 growth stages. The wavelet features for each spectral band were calculated across different scales using the CWA method, and their relationships with NNI, plant dry matter (PDM), and plant N concentration (PNC) were analyzed using four regression models. The results showed that NNI varied from 0.61 to 1.19 across different N treatments during the V6 to V12 stages, and the Mexican Hat wavelet was identified as the most suitable mother wavelet, achieving an *R* ^²^ value of 0.73 for NNI assessment. The wavelet features derived from the Mexican Hat wavelet were effective in estimating NNI, PDM, and PNC under varying N treatments, with the most sensitive wavelet features identified as 745 nm at scale 7 for NNI, 819 nm at scale 5 for PDM, and 581 nm at scale 6 for PNC using linear regression models. The direct and indirect methods for NNI estimation were compared using independent field data sets. Both methods proved valid to predict NNI in summer maize, with relative root mean square errors of 10.8% for the direct method and 13.4% for the indirect method. The wavelet feature at 745 nm, scale 7, from the direct method (NNI = 0.14 WF (745 nm, 7) + 0.3) was found to be simpler and more accurate for NNI calculation. These findings provide new insights into the application of the CWA method for precise spectral estimation of plant N status in summer maize.

## 1 Introduction

Nitrogen nutrition index (NNI) is a well-known tool that can diagnose crop nitrogen (N) status accurately and has shown potential for estimating crop yield and quality, plant N uptake and partition, photosynthesis capacity, and so on (Ata-Ul-Karim et al., 2016, 2017; Dordas, 2017; Hu et al., 2014). Its calculation is the ratio between actual plant N concentration (PNC) and plant critical N concentration (N_c_) based on the same plant dry matter (PDM) (Lemaire et al., 2008). The NNI provides a quantitative measure of the N status of crops, which is essential to optimize N fertilizer use in improving crop yield and quality. The NNI is increasingly used to assess and manage crop N requirements more accurately. However, the current methods to determine NNI have certain limitations, such as reliance on labor-intensive field sampling and variability in measurements due to environmental factors (Ziadi et al., 2010). In order to reduce the determination time of NNI, previous studies have reported some rapid and non-destructive methods to assess NNI based on chlorophyll meter and remote sensing (Zhao et al., 2018).

The assessment of plant N status is an important application of remote sensing in the agriculture sector; its application is based on the analysis of canopy spectral reflectance on crops (Li et al., 2014). Many spectral indices have been developed to estimate crop growth indices (PDM, PNC, leaf area index, and chlorophyll and pigment content) to monitor and diagnose crop N status (Schlemmer et al., 2013; Gnyp et al., 2014; Jay et al., 2016). However, these growth indices are difficult to use in estimating the extent of plant N deficit qualitatively and quantitatively due to the lack of a critical value during crop growth. NNI is better to use than a single growth index (PDM, PNC, and so on) to estimate plant N status qualitatively and quantitatively since it contains two growth indices (PDM and PNC) and is based on the theory of N_c_ dilution (Lemaire et al., 2008). At present, a few studies have developed some empirical models to estimate NNI value using canopy spectral reflectance; the estimation method was classified into two types: direct method and indirect method. The two methods with traditional spectral indices are indeed mentioned, but to fully understand their impact on practical applications, it is important to delve deeper into the specific reasons for their instability in different environments. Traditional spectral indices often suffer from sensitivity to variations in soil background, atmospheric conditions, and sensor angles, which can lead to inconsistencies in the data and unreliable results.

The direct method is such that spectral indices construct the relationship with NNI directly. Mistele and Schmidhalter (2008) utilized the red edge inflection point (REIP) to directly estimate the NNI of winter wheat. The indirect method involves estimating PDM and PNC using canopy sensing technologies to calculate NNI. Cao et al. (2013) demonstrated that while PDM could be reliably estimated using spectral indices, the performance of PNC estimation using Crop Circle ACS-470 was less satisfactory due to a lower *R* ^²^ value. Previous studies have shown that the accuracy of NNI estimation varies across different crops and environmental conditions. The spectral indices used in these studies are typically designed to estimate specific growth parameters, such as PDM, PNC, or leaf area index, rather than being exclusively tailored for NNI assessment. Additionally, the structure of some spectral indices is quite complex, requiring reflectance measurements at multiple points along the spectral curve. A simple and exclusive spectral index is needed to estimate NNI.

Recently, continuous wavelet analysis (CWA) is considered as an emerging spectroscopy tool for the quantitative analysis of biochemical constituent concentrations from leaf and canopy spectral reflectance (Cheng et al., 2011, 2014a). CWA decomposes the reflectance spectra into a series of scale components, and every component has the same length as reflectance spectrum and is composed of wavelet features as a function of wavelength and scale (Li et al., 2018). This analysis has shown the potential to estimate water content (Cheng et al., 2011; Cheng et al., 2014a; Ullah et al., 2012), chlorophyll content (Liao et al., 2013; Li et al., 2017; He et al., 2018), and nitrogen content (Li et al., 2018) from leaf and canopy reflectance spectra. The CWA method can offer greater stability and accuracy by reducing sensitivity to such environmental factors. By focusing on the specific spectral features of the target parameter, CWA can provide more consistent and reliable measurements, even under varying conditions. This robustness in diverse environments underscores the practical benefits of adopting the CWA method in modern agricultural applications.

Unlike traditional spectral indices, which often struggle with the complexity and volume of hyperspectral data, CWA excels in decomposing and analyzing these data sets. It allows for the extraction of meaningful features across multiple scales, leading to more accurate and nuanced interpretations of the data (Kaewpijit et al., 2003). To date, there is no attempt to analyze systematically the relationship between the wavelet feature of reflectance spectrum from visible light to near infrared and NNI using CWA method. The hypothesis of this study was that CWA could be used to assess the N status of summer maize. Therefore, the objectives of this study are to compare different mother wavelets in CWA method to determine the most suitable mother wavelet, to develop wavelet features based on a mother wavelet across a series of scales and wavelengths (visible light to near infrared), to identify the most accurate of the wavelet features to estimate NNI based on comprehensive analysis, to construct optimum regression models between wavelet features and NNI using the direct and indirect methods during the V6 to V12 growth stages of summer maize, and to validate the developed regression models of the direct and indirect methods to establish the most appropriate way for NNI estimation. This study will provide a new technical support to diagnose plant N status based on CWA method to analyze the canopy reflectance spectra of summer maize.

## 2 Materials and methods

### 2.1 Experiment design

**Figure 1:**
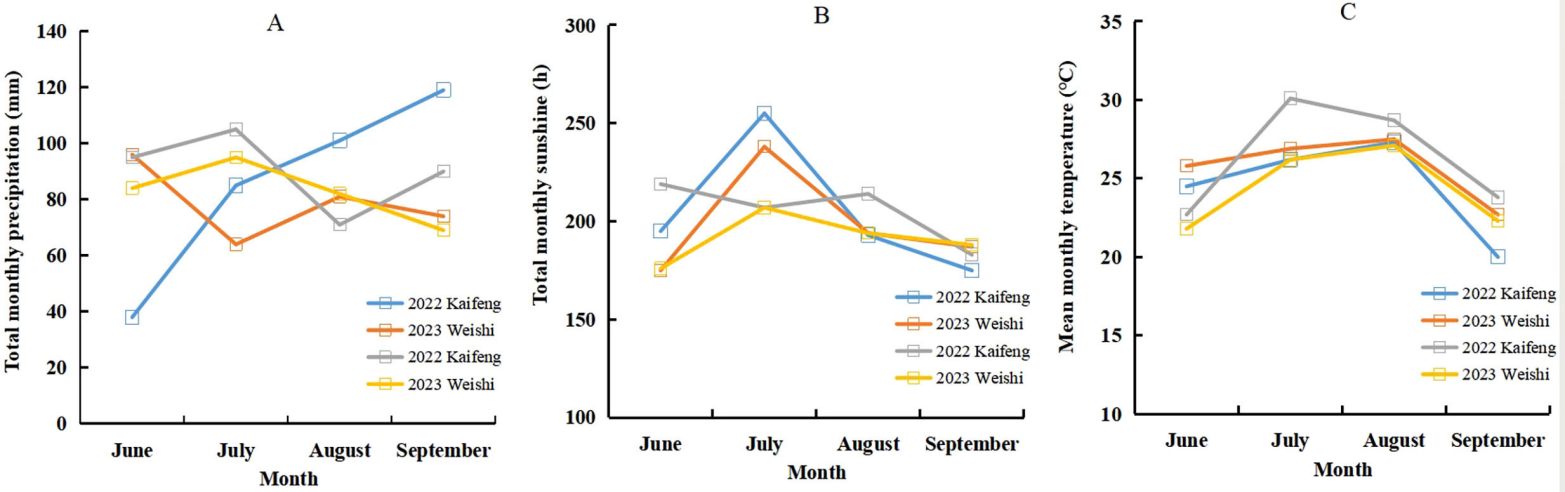
Total monthly precipitation (**A**, mm), total monthly sunshine (**B**, h) and mean monthly temperature (**C**, ℃) during 2022 and 2023 seasons of summer maize.

During the 2022 and 2023 seasons, field experiments of summer maize were carried out at Kaifeng and Weishi in China, respectively. These experiments included four cultivars of summer maize and five N application treatments. Detailed information about the series of field experiments is shown in **Table 1**. The 0–20-cm soil samples were sampled before the planting of summer maize and then air-dried, and sieved to measure total N, Olsen-P, NH_4_OAc-K^+^, and organic matter (Nelson and Sommers, 1982; Bremner and Mulvancy, 1982; Olsen et al., 1954; van Reeuwijk, 1992). Weather information of the season of summer maize is shown in **Figure 1**. Randomized complete block design was used in every experiment with three replicates. The size of each plot was 60 m^2^ (6 m × 10 m) in every field experiment. The total N fertilizer was divided into base fertilizer (50%) and top-dressing fertilizer (50%), which were applied before sowing and at the V6 stage, respectively. Adequate amounts of phosphate fertilizer (triple superphosphate) and potash fertilizer (potassium chloride) were applied into the soil before sowing. The planting density for all the experiments was 60,000 plants ha^-1^, with a row spacing of 60 cm. Moreover, 40 mm was irrigated into the field to ensure the emergence of summer maize. During the growth progress of summer maize, the irrigation amount ranged from 250 to 350 mm, and the fertilization timings were in mid-August during the 2022 and 2023 growth seasons. Additional crop management was consistent with local agriculture production. There was no obvious water, disease, and pest stress during growth season of summer maize. The amount of N input was the only limiting factor during the process of the field experiments.

**Table 1:** Characteristics of the six experiments in this study.

Experiment No.	Cultivar	Soil characteristics	N(kg N ha^–1^)	Sampling stage
Experiment 1	Xundan20	Type: sandy soil	0 (N0)	V6
(2022 Kaifeng)	(XD20)	Organic matter: 9.3 g kg^–1^	80 (N1)	V9
		Total N: 0.62 g kg^–1^	160 (N2)	V12
		Olsen-P: 10.5 mg kg^–1^	240 (N3)	
		NH_4_oAc-K^+^: 72.5 mg kg^–1^	320 (N4)	
Experiment 2	Zhengdan958	Type: light loam soil	0 (N0)	V6
(2022 Weishi)	(ZD958)	Organic matter: 11.73 g kg^–1^	75 (N1)	V9
		Total N: 0.58 g kg^–1^	150 (N2)	V12
		Olsen-P: 34.52 mg kg^–1^	225 (N3)	
		NH_4_oAc-K^+^: 75 mg kg^–1^	300 (N4)	
Experiment 3	Denghai661	Type: sandy soil	0 (N0)	V6
(2023Kaifeng)	(DH661)	Organic matter: 8.4g kg^–1^	75 (N1)	V9
		Total N: 0.52 g kg^–1^	150 (N2)	V12
		Olsen-P: 11.3 mg kg^–1^	225 (N3)	
		NH_4_oAc-K^+^: 71.5 mg kg^–1^	300 (N4)	
Experiment 4	Denghai605	Type: light loam soil	0 (N0)	V6
(2023Weishi)	(DH605)	Organic matter: 11.2 g kg^–1^	90 (N1)	V9
		Total N: 0.48 g kg^–1^	180 (N2)	V12
		Olsen-P: 21.52 mg kg^–1^	270 (N3)	
		NH_4_oAc-K^+^: 54.23 mg kg^–1^		

### 2.2 Field sampling and measurement

To obtain a representative plant sample, six plants were destructively sampled at the V6, V9, and V12 stages by randomly cutting in the middle of each plot. All of these samples were oven-dried at 80°C to a constant weight and then weighed and ground for chemical analysis later. PNC was determined using the traditional Kjeldahl method (Bremner and Mulvancy, 1982). Canopy spectral reflectance was measured using a portable spectrometer (FieldSpec Handheld 2; Analytical Spectral Devices (ASD), USA) at 10 A.M. and 14 P.M. local time under cloudless conditions. The canopy reflectance was calculated through the calibration of measurements of dark current and a white spectrum on the reference panel with known reflectance properties. The spectrometer covers the 325–1,075-nm (visible light to near-infrared) spectral range, with 1.4-nm sampling interval and 25°field of view. The data of spectral reflectance was re-sampled to 1-nm bandwidth using a self-driven interpolation method of this machine and then saved. Each measurement was taken randomly at five sites in each plot at a height of 50 cm above the plant canopy; scans of 10 times were collected in each site and then calculated as an average curve to represent the canopy reflectance spectra of each plot. The calibration of the spectrometer was taken every 15 min to correct potential effects caused by changes in the external environment. Due to machine noise and the external environment, the spectral value varied irregularly at the 906- to 1,075-nm region. In this study, 325–905 nm was used to develop the relationships between spectral value and NNI. Canopy reflectance was collected at the V6, V9, and V12 stages of summer maize. These stages are the critical time windows for top-dressing N fertilizers on summer maize.

### 2.3 Calculation of nitrogen nutrition index

NNI is calculated based on the N_c_ dilution curve of summer maize. This curve has been developed by Plénet and Lemaire (2000) and shown in (Equation 1). The calculation of NNI is listed in (Equation 2).


(1) $$N_{c}=3.4PDM^{-0.37}$$

(2) $$NNI=\frac{N_{a}}{N_{c}}$$

where PDM is plant dry matter (t ha^-1^), N_c_ is plant critical N concentration (%), and N_a_ is plant actual N concentration (%).

### 2.4 Wavelet analysis

Wavelet analysis is an efficient signal processing tool that can decompose the original signal into multiple scales, which has been successfully applied to hyperspectral data for dimensionality reduction (Bruce et al., 2001). Wavelet transform is a very important step to analyze hyperspectral data in wavelet analysis. Wavelet transform includes two variations: discrete wavelet transform (DWT) and continuous wavelet transform (CWT) (Cheng et al., 2014a). Cheng et al. (2011) recommended the CWT method to analyze the relationships between hyperspectral data and agronomy variables.

**Figure 2:**
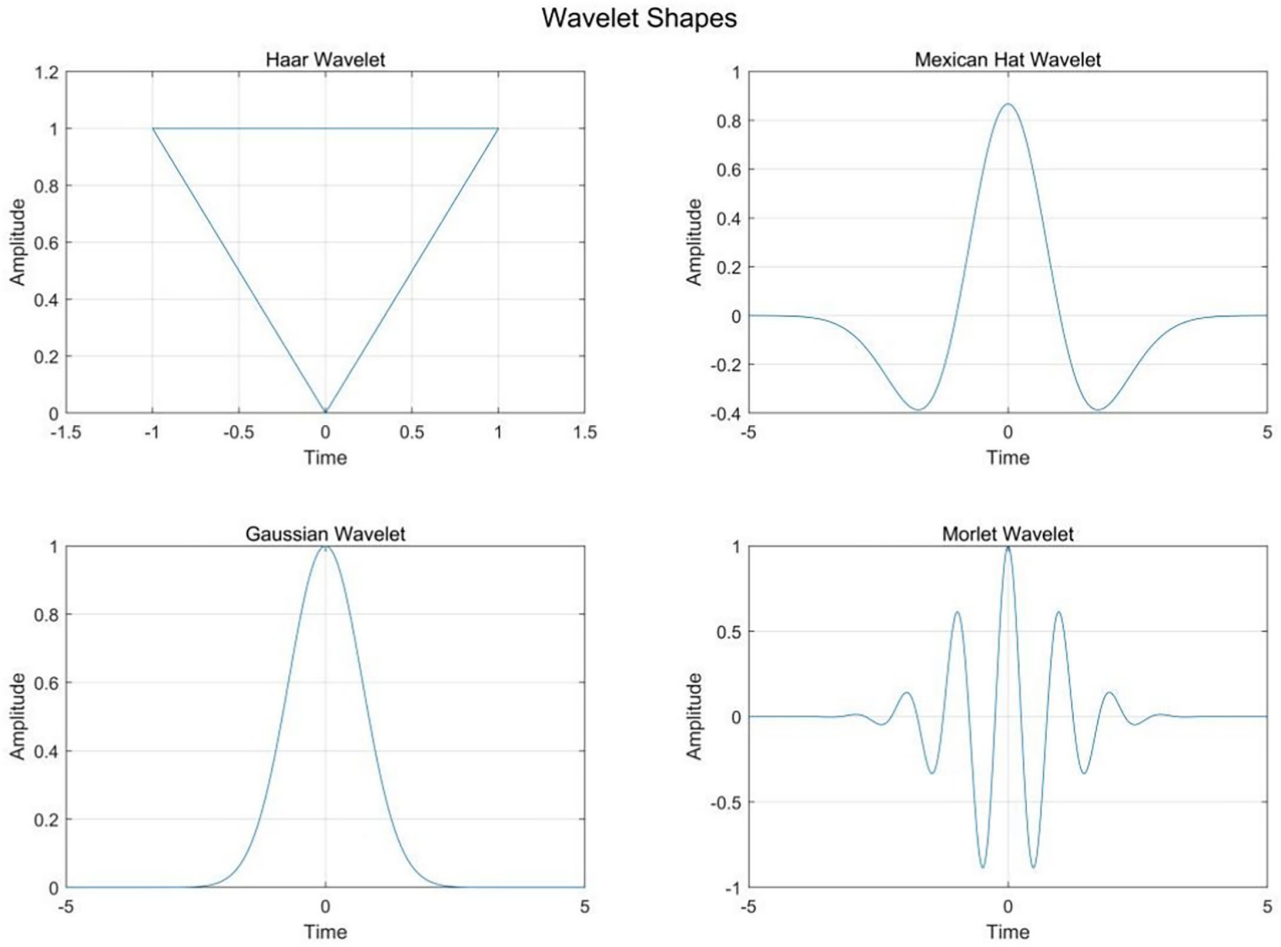
The shape of four different mother wavelets.

Continuous wavelet transform is a linear operation that transforms the convolution of reflectance spectra f(λ) with a scaled and shifted mother wavelet. The mother wavelet is expressed as shown below (Equation 3):


(3) $$\varphi_{a,b}(\lambda )=\frac{1}{\sqrt{a}}\varphi (\frac{\lambda -b}{a})$$

where ϕ(λ) is the mother wavelet function, *a* is the scaling factor representing the width of the mother wavelet, which can be comparable with the width of an absorption feature, and *b* is the shifting factor determining the position, which denotes the band position (325 to 905 nm) of the hyperspectral curve. The result of CWT is calculated as shown below (Mallat, 1991) (Equation 4):


(4) $$W_{f}(a,b)=\langle f,\varphi_{a,b}\rangle =\int_{-\infty }^{+\infty }f(\lambda )\varphi_{a,b}(\lambda )d\lambda$$

where W_f_(a,b) is the wavelet feature (coefficient) that is the inner product of wavelets and spectrum reflectance. In this study, Mexican Hat, Gaussian Hat, Morlet Hat, and Haar Hat are used as the mother wavelet bases. **Figure 2** shows the shape of the four mother wavelets, which was used to compare which mother wavelet can best represent the relationship with NNI (Ngui et al., 2013). All CWT procedures are completed by means of the wavelet toolbox of MATLAB 7.

**Figure 3:**
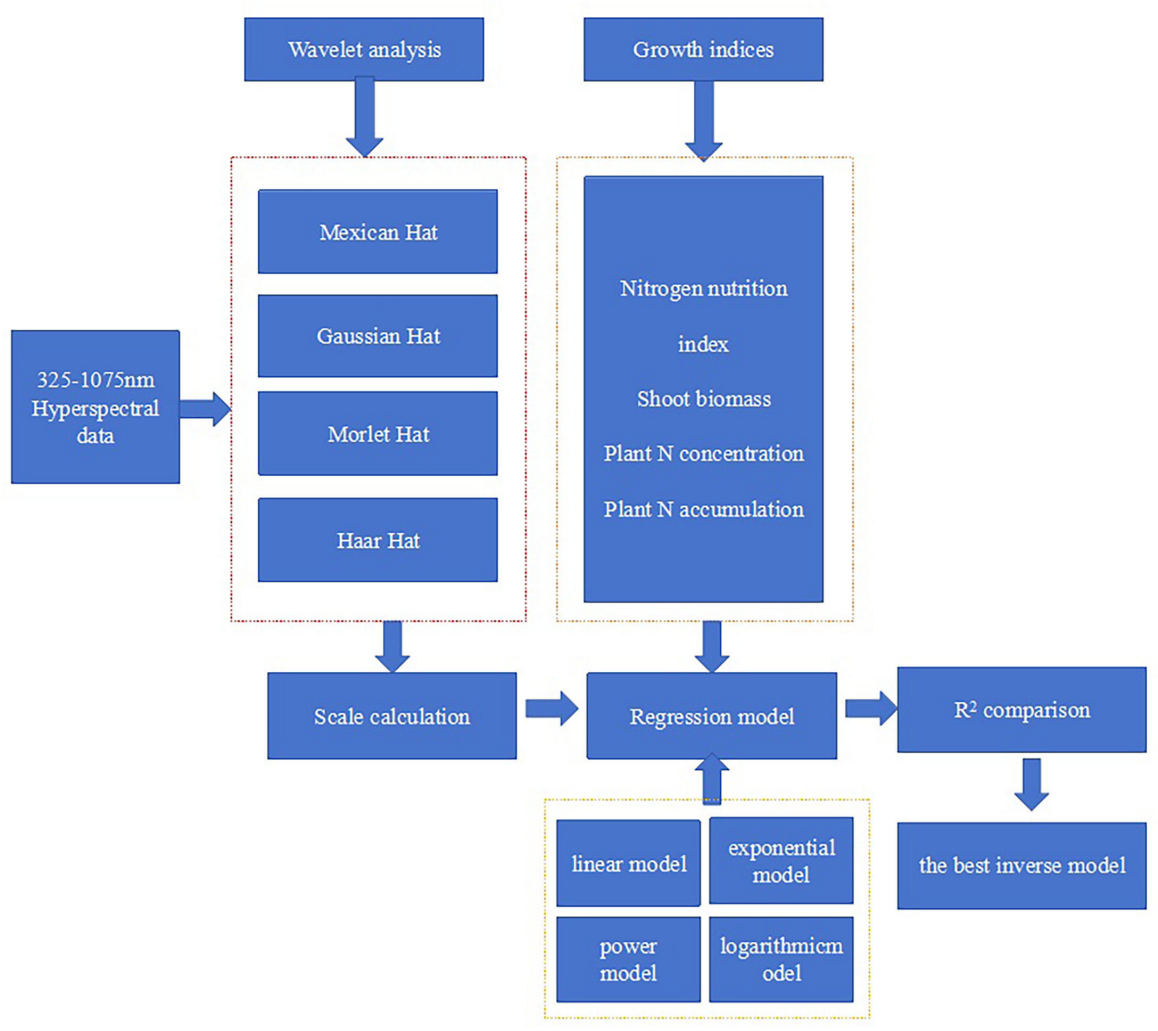
Flowchart for constructing a nitrogen nutrition index inversion model based on wavelet analysis.

The selection method of the sensitive wavelet feature is divided by four main steps. At the first step, the average *R* ^2^ values were used to compare the difference between NNI and wavelet features under different mother wavelet conditions. At the second step, the spectral curve of each treatment is imported into the continuous wavelet 1-D function of the wavelet toolbox in MATLAB 7. The wavelet coefficient of every reflectance spectra was calculated as a function of wavelength (325 to 905 nm) and scale (Power 2 Mode; power coefficient is 10). A scalogram of wavelet power with dimensions of power, wavelength, and scale is shown using the analysis system of continuous wavelet 1-D function. At the third step, the wavelet coefficient of the scalogram was read progressively within the range of 325–905-nm wavelength and 2^1^ to 2^10^ scale; every wavelet coefficient is regressed with NNI, including linear, power, and logarithmic and exponential types. The contour map of determination coefficients (*R* ^2^) is plotted according to the change of *R* ^2^ values. This step is completed with a self-programmed software by using MATLAB 7. At the fourth step, the most sensitive region and wavelet coefficient (wavelength in nanometers, scale) were determined by the maximum *R* ^2^ value based on the scalogram plot. The specific technical flowchart is shown in **Figure 3**. The regression figure between the optimum wavelet coefficient and NNI was plotted using Microsoft Excel (Microsoft Corporation, Redmond, WA, USA).

### 2.5 Statistical analysis

Univariate multivariate analysis of variance is used to analyze the difference of PNC, plant nitrogen uptake, PDM, and NNI using SPSS v.13 software package (SPSS Inc., Chicago, IL, USA). The fix factors are season, cultivar, and N treatment in the analysis process. The significance level was preset as *P <*0.05, *P <*0.01, and *P <*0.001 for all hypothesis testing. The calibration data sets from experiments 1 and 3 are used to develop the relationships between NNI and wavelet feature, and the validation data sets from experiments 2 and 4 are used to validate these developed relationships. The statistical parameters coefficient of determination (*R* ^2^), root mean square error of calibration (RMSEC), and relative error of calibration (REC) are used to evaluate goodness of fit, and the other statistical parameters relative root mean square root (RRMSE), root mean square error of prediction (RMSEP), and relative error of prediction (REP) were used to assess prediction abilities and stability. Microsoft Excel (Microsoft Corporation, Redmond, WA, USA) was used to calculate the parameters. The calculation equations of *R* ^2^, RMSEC, REC, RMSEP, RRSME, and REP are shown as follows (Equations 6–11):


(5) $$R^{2}=1-\frac{\sum_{i=1}^{n}{\left(O_{i}-P_{i}\right)}^{2}}{\sum_{i=1}^{n}{\left(O_{i}-\overset{-}{O}\right)}^{2}}$$

(6) $$RMSEC=\sqrt{\frac{\sum_{i=1}^{n}{\left(O_{i}-O_{i}^{'}\right)}^{2}}{n}}$$

(7) $$RMSEP=\sqrt{\frac{\sum_{i=1}^{n}{\left(O_{i}-P_{i}\right)}^{2}}{n}}$$

(8) $$RRMSE=\frac{RMSEP}{\overset{-}{O}}\times 100\%$$

(9) $$REC=\frac{\sum_{i=1}^{n}\left|O_{i}-O_{i}^{'}\right|}{n\times \overset{-}{O}}\times 100\%$$

(10) $$REP=\frac{\sum_{i=1}^{n}\left|O_{i}-P_{i}\right|}{n\times \overset{-}{O}}\times 100\%$$

where P_i_ is the estimated value from the regression model, O_i_ is the observed value, Ō is the average value of all observed values, O’ is the calculation value of the regression model, and n is the number of samples. The higher *R^2^* value and the lower values of RMSEC and REC are considered as a higher goodness of fit between wavelet feature and NNI, and the lower values of RMSEP, REP, and RRMSE are considered as a higher predicted accuracy of the developed models.

### 2.6 Spectral indices

Some spectral indices have been used to estimate the NNI values of different crops. In this study, three commonly used spectral indices (**Table 2**) were chosen to test their usefulness to estimate the NNI of summer maize. Red edge inflection point (REIP-LI) was used by Mistele and Schmidhalter (2008) to assess the NNI of winter wheat. MTCI was proposed by Chen (2015) to relate canopy reflectance with the NNI of winter wheat. Modified red edge simple ratio index (mSR_705_) was recommended by Liu et al. (2018) to estimate the NNI of winter oilseed rape.

**Table 2:** Spectral indices for predicting nitrogen nutrition index.

Index	Name	Formula	Developed by
REIP-LI	Red edge inflection point	$700+40\frac{(R_{670}+R_{780})/2-R_{700}}{R_{740}-R_{700}}$	Mistele and Schmidhalter (2008)
MTCI	MERIS Terrestrial Chlorophyll Index	$\frac{R_{750}-R_{710}}{R_{710}-R_{680}}$	Chen (2015)
mSR_705_	Modified Red Edge Simple Ratio Index	$\frac{R_{750}-R_{445}}{R_{705}+R_{445}}$	Liu et al. (2018)

## 3 Results

### 3.1 Variance analysis of plant nitrogen concentration, plant biomass, plant nitrogen uptake, and nitrogen nutrition index

In this study, there was no significant difference of plant N concentration, plant N uptake, plant biomass, and NNI across cultivars (XD20 and DH661) and seasons (2022 and 2023). PNC, PDM, NNI, and plant N uptake differed significantly by N application rate at the *P <*0.001 level (**Table 3**). The effect on PNC, PDM, NNI, and plant N uptake was shown non-significantly under the interaction of season × cultivar condition; however, the effects on PDM and plant N uptake (*P* < 0.001) and PNC and NNI (*P* < 0.01) were observed significantly under the interaction of season × N treatment and cultivar × N treatment condition. There was a significant effect (*P* < 0.05) on PNC, NNI, PDM, and plant N uptake (*P* < 0.01) under the season × cultivar × N treatment condition. These parameters have shown a large variability under different N conditions, which made a good data set to develop the relationship between NNI and reflectance spectra.

**Table 3:** Variance analysis of plant nitrogen concentration, plant dry matter, plant nitrogen uptake and nitrogen nutrition index affected by season, cultivar, nitrogen treatments and their interactions.

Source	PNC (%)	Plant N uptake (kg ha^-1^)	PDM (t ha^-1^)	NNI
Cultivar(C)	NS	NS	NS	NS
Season(S)	NS	NS	NS	NS
N treatment (N)	***	***	***	***
S×C	NS	NS	NS	NS
S×N	***	**	***	**
C×N	***	**	***	**
S×C×N	**	*	**	*

NS represents no significant at 0.05 probability level.* Represents significant at 0.05 probability level.** Represents significant at 0.01 probability level.*** Represents significant at 0.001 probability level.

### 3.2 Change of nitrogen nutrition index across different nitrogen treatments and growth stages

**Figure 4:**
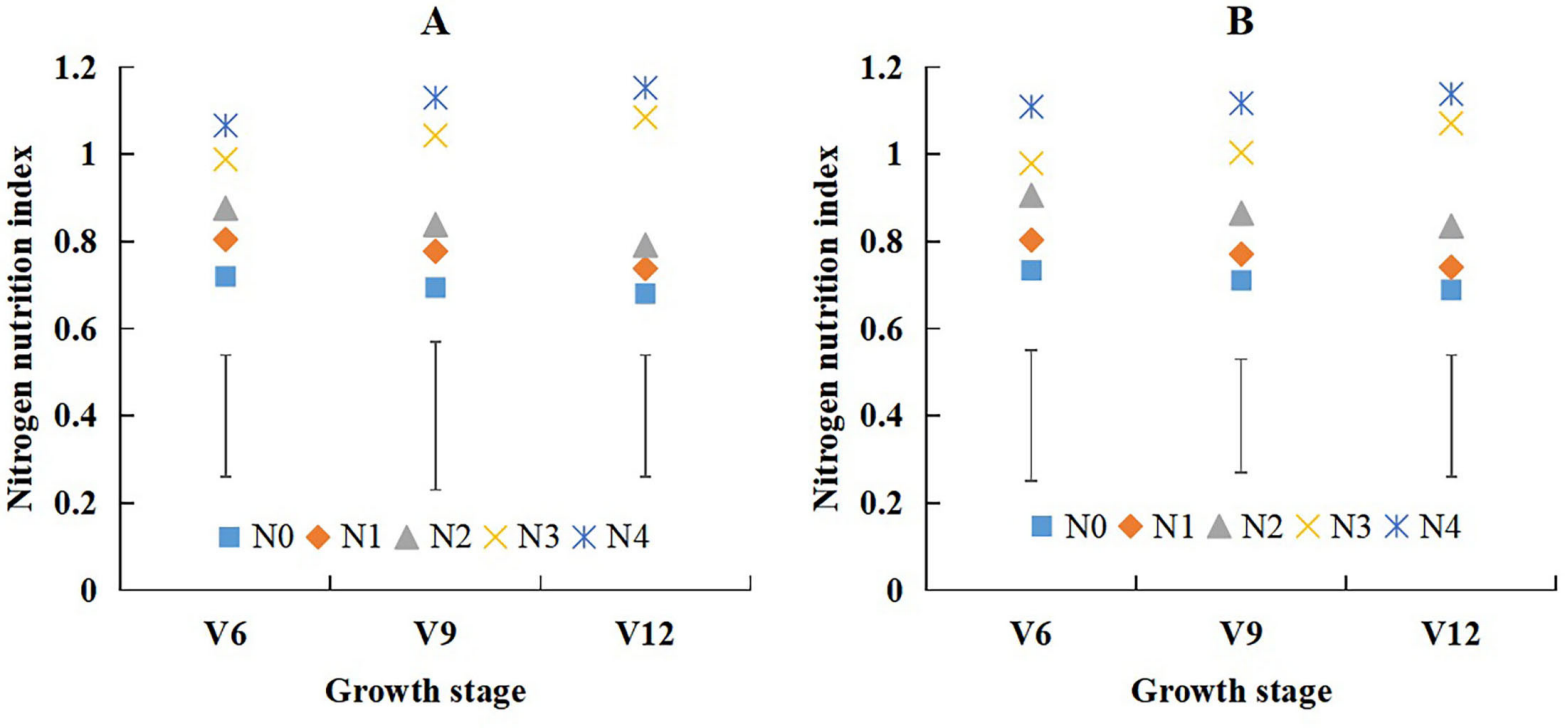
The change of nitrogen nutrition index across different nitrogen treatments from V6 to V12 growth stages of summer maize during 2022 and 2023 seasons (**(A)**: 2022 XD20; **(B)**: 2023 DH661).Vertical bars represent the value of least significant difference (P<0.05) for each growth stage.

There was significant difference of nitrogen nutrition index (NNI) under different N treatments (**Figure 4**). NNI increased with the application of N fertilizer, NNI values of XD20 ranged from 0.68 to 1.15 (**Figure 4A**), and NNI values of DH661 ranged from 0.69 to 1.14 (**Figure 4B**). The NNI values of the two cultivars were lower than those at the N0, N1, and N2 treatments, and the NNI values were nearly equal to those at the N3 treatments and were higher than those at the N4 treatments. The NNI values decreased gradually from the V6 to V12 stages of summer maize at the N0, N1, and N2 treatments; however, the NNI values increased gradually at the same stages of summer maize at the N3 and N4 treatments.

### 3.3 Change of canopy spectral reflectance across different N treatments and growth stages

**Figure 5:**
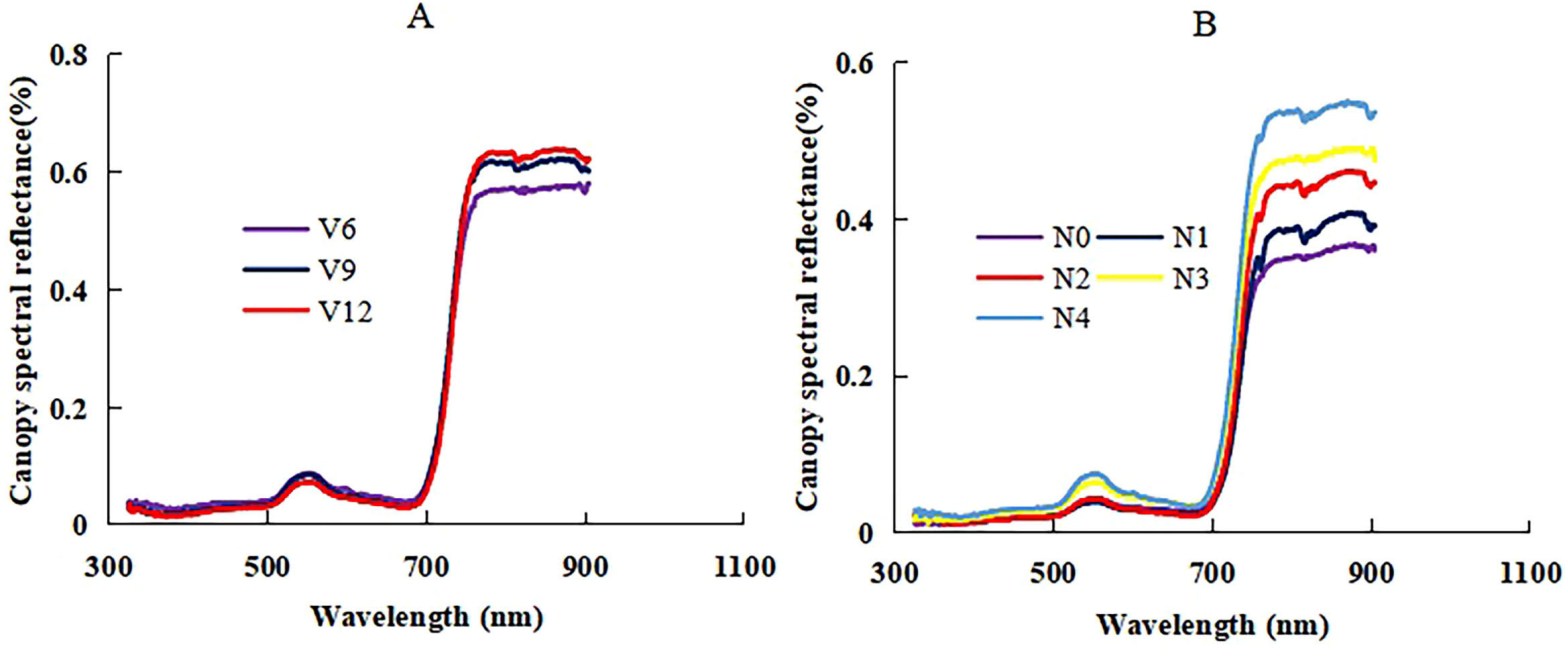
The change of canopy reflectance spectra across V6 to V12 growth stages (**(A)**: XD20 N3 treatment of 2022 season) and N0 to N4 nitrogen treatments (**(B)**: DH661 V6 stage of 2023 season) of summer maize.

In this study, the canopy spectral reflectance of summer maize increased with the growth process in the near-infrared bands, but the reflectance was not significantly different across the V6 to V12 stages in the visible bands (**Figure 5A**). N application had a significant influence on the change of canopy spectral reflectance (**Figure 5B**); the trend was similar to the growth stage. The main difference of the reflectance also existed in the near-infrared bands. The near-infrared bands could better show the effect of growth stages and N application to the canopy spectral reflectance of summer maize. The change of canopy spectral curves across different growth stages and N applications provided a basic support to analyze and develop empirical relationships between NNI and the canopy reflectance spectra of summer maize.

### 3.4 Performance of four mother wavelets with nitrogen nutrition index, shoot biomass, plant N concentration, and plant N accumulation

**Figure 6:**
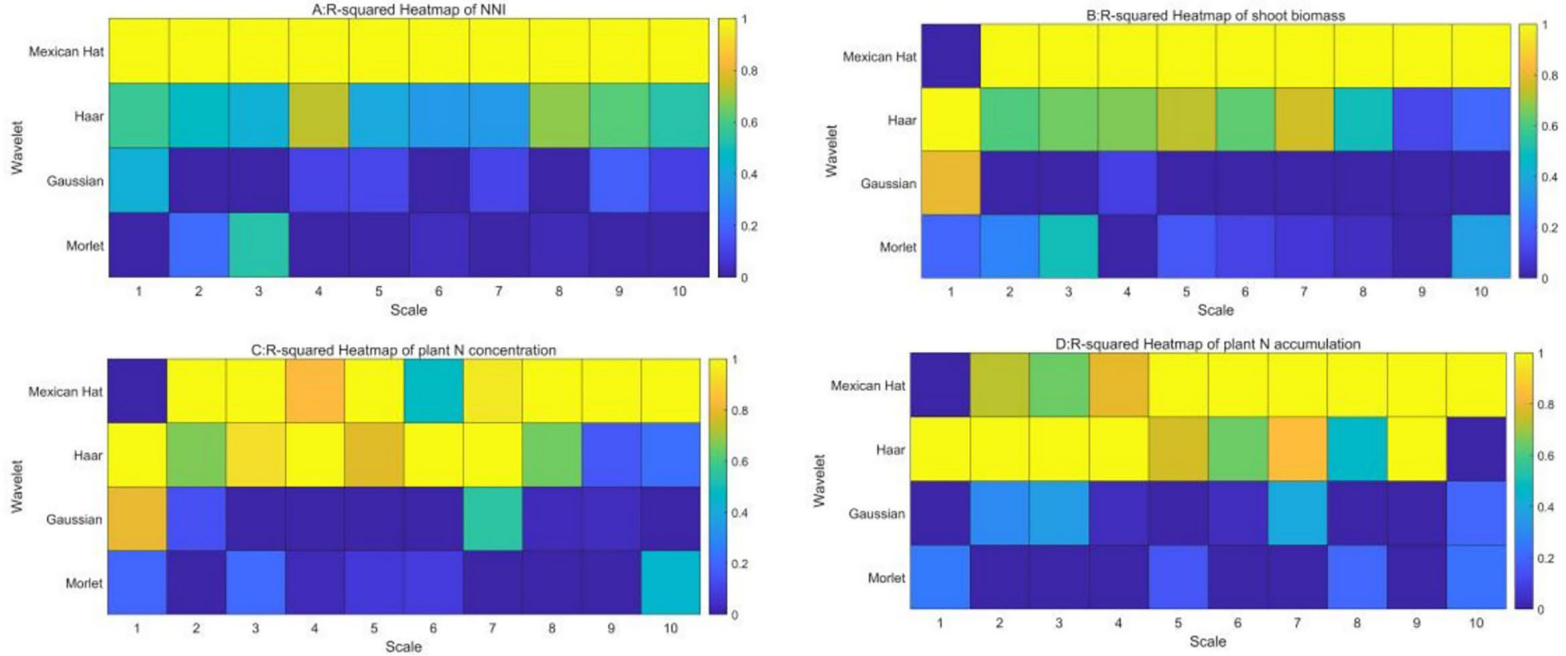
The determination coefficient of four mother wavelets with nitrogen nutrition index **(A)**, shoot biomass **(B)**, plant N accumulation **(C)** and plant N concentration **(D)**.

The relationships between four mother wavelets and nitrogen nutrition index, shoot biomass, plant N concentration, and plant N accumulation were developed across different average spectral values. The performance of the four mother wavelets is shown in **Figure 6**. The result indicated that the values of the determination coefficient (*R* ^2^) of NNI, shoot biomass, plant N concentration, and plant N accumulation with Mexican Hat were significantly higher than those of the three other mother wavelets across 1 to 10 scales. The *R* ^2^ values from Mexican Hat ranged from 0.5 to 0.7 about NNI, from 0.35 to 0.75 about shoot biomass, from 0.32 to 0.82 about plant N concentration, and from 0.28 to 0.78 about plant N accumulation across 1 to 10 scales, which was, on average, 30% to 50% higher than those of the three other methods (**Figure 6**). Therefore, the Mexican Hat could be considered as the best mother wavelet to assess NNI and growth indices in summer maize.

### 3.5 Estimation model of nitrogen nutrition index using the direct method

**Figure 7:**
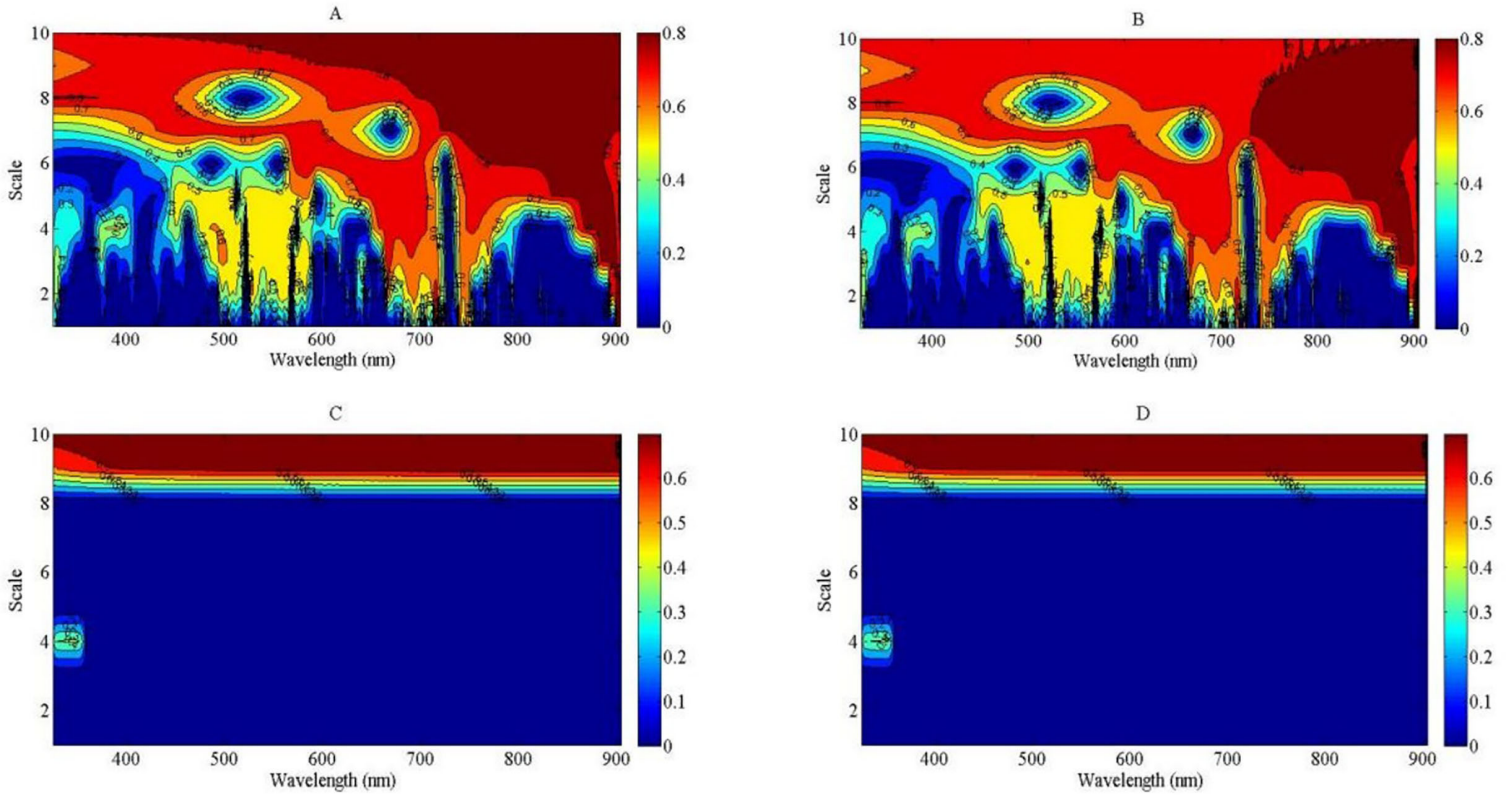
The contour maps of determination coefficient for the linear, exponential, power and logarithmic regression types between nitrogen nutrition index (NNI) and wavelet feature from 325 to 905 nm. **(A)**: *R^2^* between NNI and wavelet feature using linear model; **(B)**: *R^2^* between NNI and wavelet feature using exponential model; **(C)**: R^2^ between NNI and wavelet feature using power model; **(D)**: *R^2^* between NNI and wavelet feature using logarithmic model.

The correlation analysis between NNI and wavelet feature was developed to select the best wavelet feature to assess NNI using linear, exponential, power, and logarithmic regression models through a direct method. All of the bands were used to construct the empirical relationships between NNI and wavelet feature, from the visible light to NIR bands (325 to 905 nm), based on continuous wavelet analysis. The results indicated that the *R* ^2^ values were higher than 0.8 at the NIR region compared with the four regression types. The *R* ^2^ values of the linear and exponential regression models were greater than those of the power and logarithmic regression models at the NIR region (**Figures 7A, B**). The *R* ^2^ values of the power and logarithmic regression models were equal to 0 at the lower scale region of the wavelet feature, which could not be used to develop the relationship between NNI and wavelet feature (**Figures 7C, D**). The higher scale of wavelet feature was more suitable to assess the NNI of summer maize from the V6 to V12 growth stages using the linear and exponential regression models. The strongest relationship between NNI and wavelet feature was observed for feature (745 nm, 7) based on the linear regression and feature (784 nm, 7) based on the exponential regression. The feature (745 nm, 7) was located among the red edge region, and the feature (784 nm, 7) occurred on the right shoulder of the red edge region. The relationships between NNI and the features (745 nm, 7) and (784 nm, 7) are shown in **Supplementary Figure S1**, respectively.

### 3.6 Estimation model of nitrogen nutrition index using the indirect method

**Figure 8:**
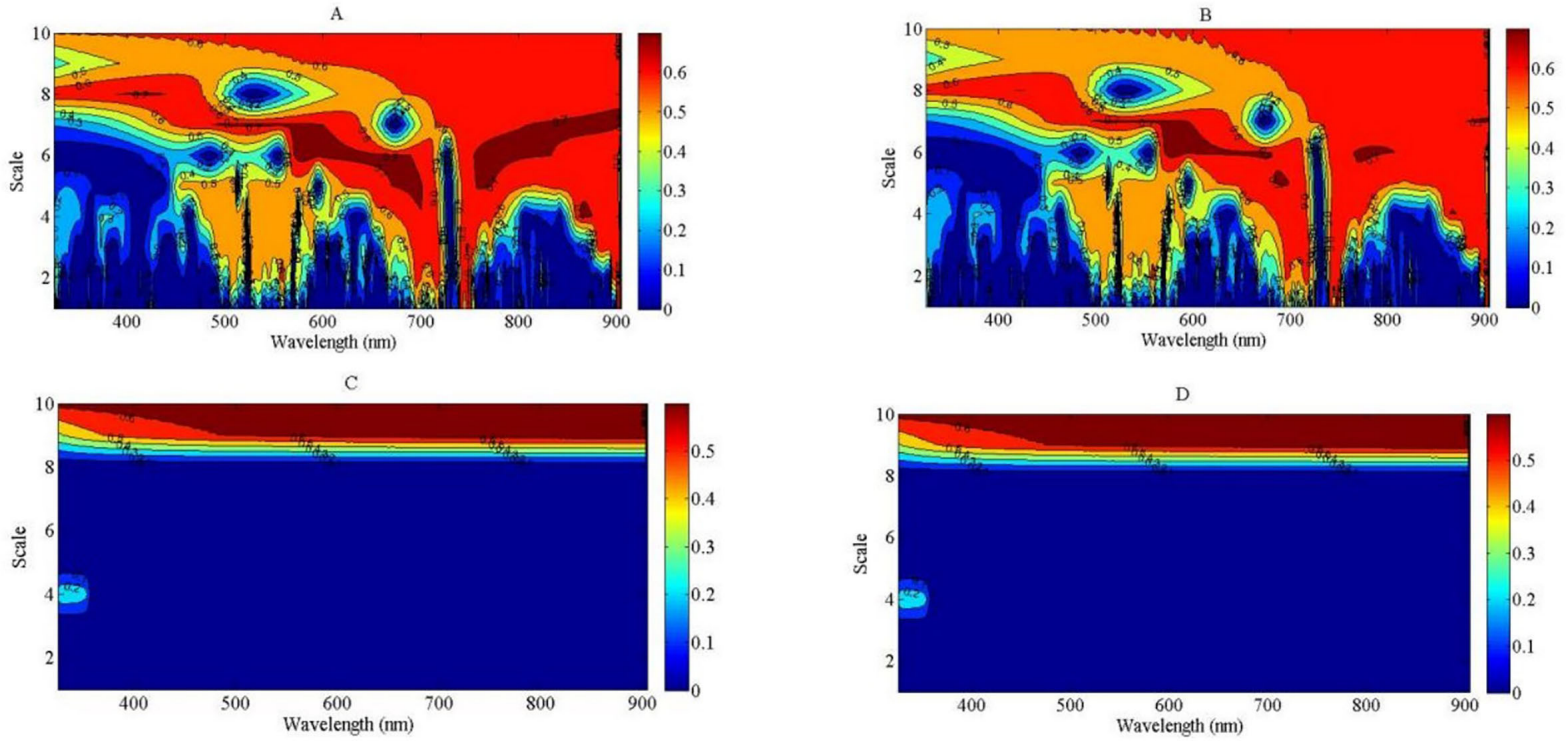
The contour maps of determination coefficient (*R^2^*) for the linear, exponential, power and logarithmic regression types between plant dry matter (PDM) and wavelet feature from 325 to 905 nm. **(A)**: *R^2^* between PDM and wavelet feature using linear model; **(B)**: *R^2^* between PDM and wavelet feature using exponential model; **(C)**: *R^2^* between PDM and wavelet feature using power model; **(D)**: *R^2^* between PDM and wavelet feature using logarithmic model.

**Figure 9:**
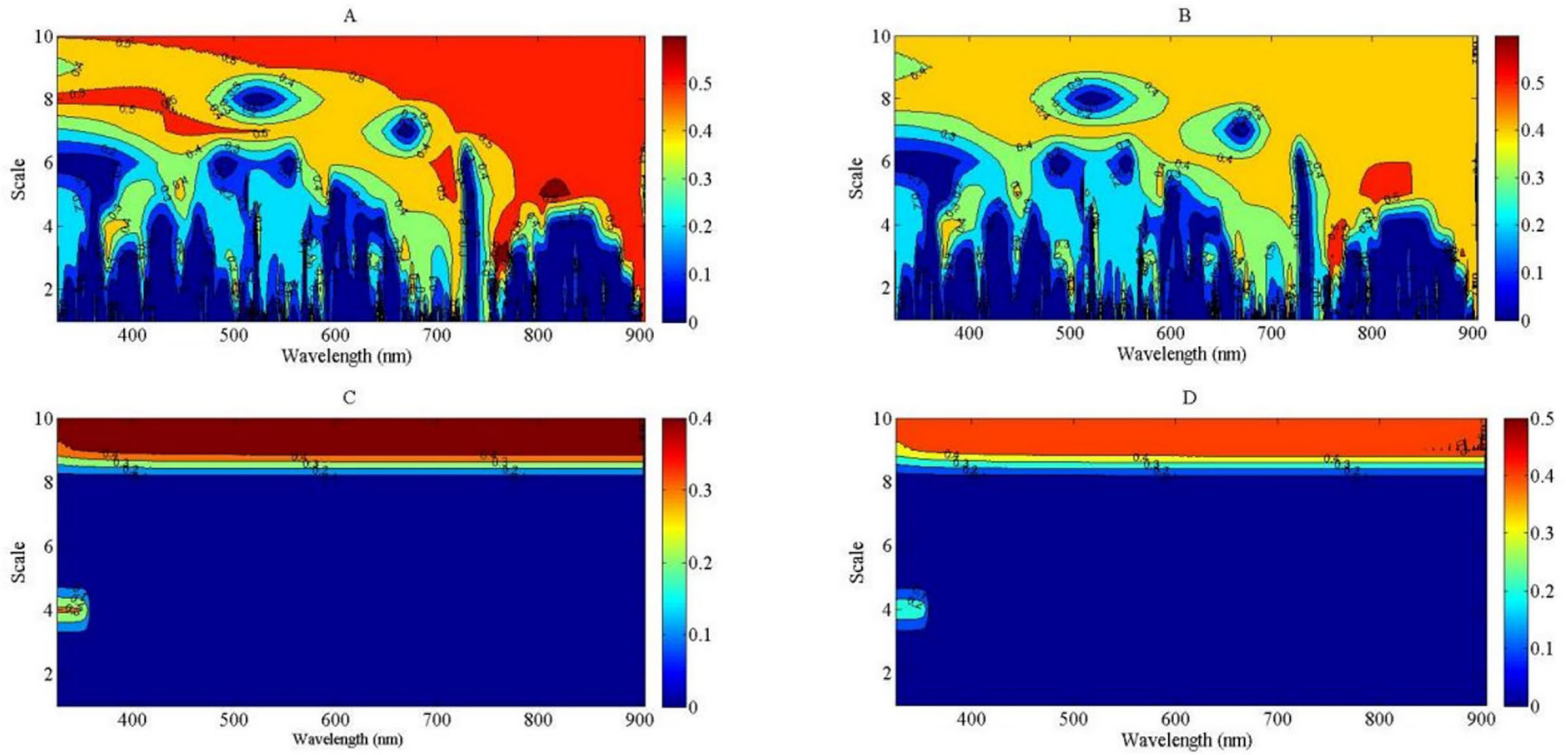
The contour maps of determination coefficient for the linear, exponential, power and logarithmic regression types between plant nitrogen concentration (PNC) and wavelet feature from 325 to 905 nm. **(A)**: *R^2^* between PNC and wavelet feature using linear model; **(B)**: *R^2^* between PNC and wavelet feature using exponential model; **(C)**: *R^2^* between PNC and wavelet feature using power model; **(D)**: *R^2^* between PNC and wavelet feature using logarithmic model.

In the indirect method, PDM was estimated using continuous wavelet analysis to calculate the NNI. The *R* ^2^ values of the regression models between PDM and wavelet feature are shown in **Figure 8**. The regression type was based on linear, exponential, power, and logarithmic regression types (**Figure 9**), respectively. The result indicated that the power and logarithmic regression types were not suitable to estimate PDM, and the *R* ^2^ value was equal to 0 at the lower scale of the wavelet feature and lower than 0.5 at the higher scale of the wavelet feature. The linear and exponential regression types were more suitable to develop the relationship between PDM and wavelet feature, and the *R* ^2^ values based on linear and exponential regression types were higher than those based on power and logarithmic regression types across different scales of the wavelet feature. The strongest relationship between PDM and wavelet feature was observed for feature (819 nm, 5) based on the linear regression and feature (782 nm, 3) based on the exponential regression. The features (819 nm, 5) and (782 nm, 3) were located at the near-infrared region. The relationships between PDM and the features (819 nm, 5) and (782 nm, 3) are shown in **Supplementary Figure S2**, respectively.

Another estimated variable was PNC to calculate NNI in the indirect method. The four regression types (linear, exponential, power, and logarithmic) were used to develop the relationship between PNC and wavelet feature based on continuous wavelet analysis. The regression performance of power and logarithmic types between PNC and wavelet feature was similar with that between PDM and wavelet feature (**Figures 8C, D**). The *R* ^2^ value was lower at the low-scale region of the wavelet feature than at the high-scale region of the wavelet feature across the two regression types, which was equal to 0 at the low scale. The regression performance of the linear and exponential types was better than that of the power and logarithmic types. The *R* ^2^ value was higher than 0.7 from the visible light to the NIR bands under the linear and exponential regression types (**Figures 8A, B**). The sensitive region between PNC and wavelet feature was greater under the linear regression type than under the exponential regression type. The optimal relationship between PNC and wavelet feature was observed for feature (581 nm, 6) under the linear regression type and feature (573 nm, 6) under the exponential regression type (**Supplementary Figure S3**). The optimal WP mainly existed at the higher region of scale. The *R* ^2^ value was slightly higher based on linear regression than exponential regression. Therefore, the PDM estimation model of feature (819 nm, 5) and the PNC estimation model of feature (581 nm, 6) were used to assess the NNI value in the indirect method. The integrated model of NNI was expressed as follows:


(11) $$NNI=\frac{-3.38WP(581nm,6)+2.1}{3.4{\left(6.59WP(819nm,5)+0.03\right)}^{-0.37}}$$

### 3.7 Validation of the estimation linear model of nitrogen nutrition index based on wavelet features

**Figure 10:**
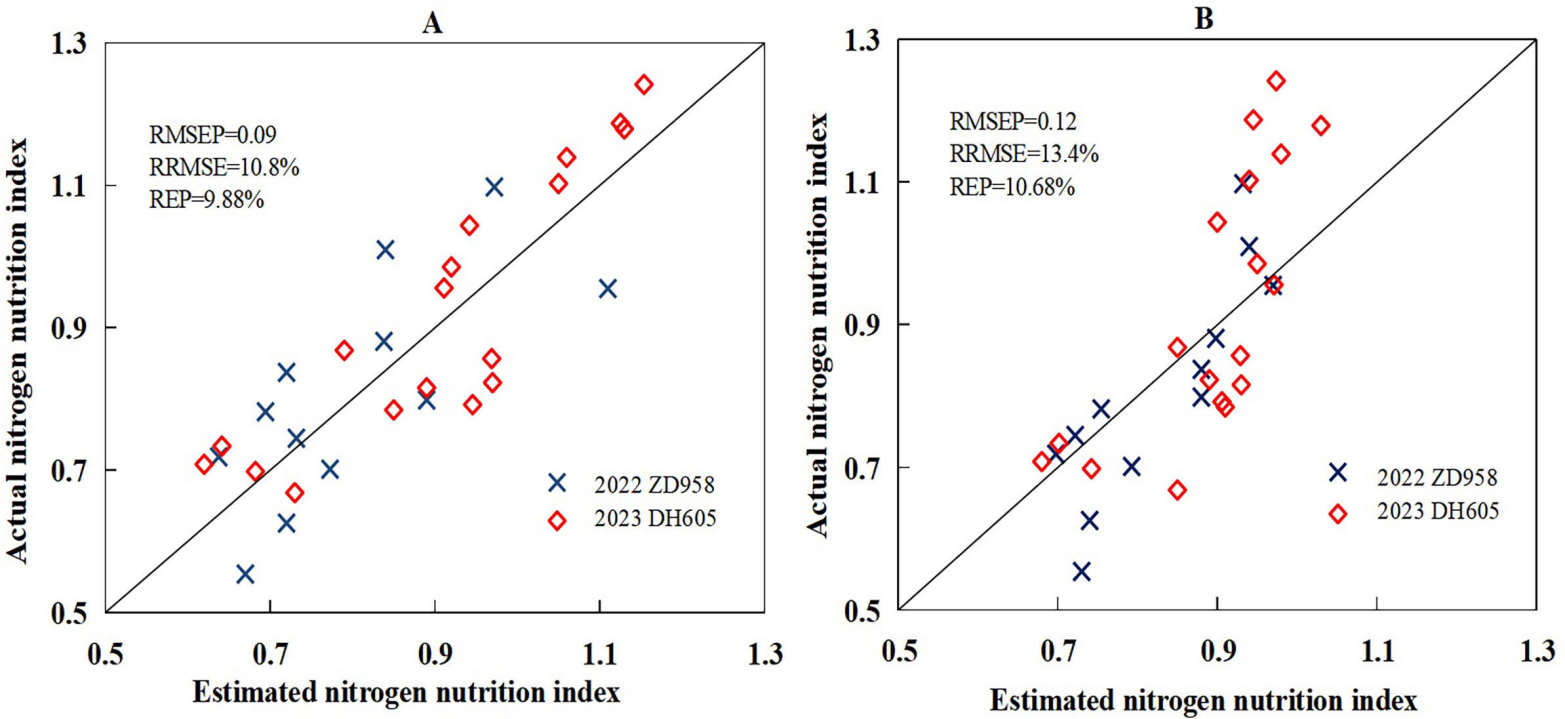
The validation result between estimated nitrogen nutrition index based on the direct method (**A**, linear) and indirect method (**B**, linear) and actual nitrogen nutrition index using the validation data set acquired from experiments 3 and 4.0.

The calibration result showed that the goodness of fit of the linear model was better than that of the exponential model, so this study chose the linear model to validate the feasibility of wavelet analysis for assessing NNI. The independent experiment data sets (experiments 3 and 4) were used to validate the newly developed regression models based on wavelet features (**Figure 10**). The result indicated that the performances of the new models were acceptable using the direct and indirect methods. In the direct method, the wavelet feature (745 nm, 7) produced an accurate prediction of NNI values, with RMSEP, RRMSE, and REP values of 0.09, 10.8%, and 9.88%, respectively. In the indirect method, (Equation 11), which included wavelet feature (581 nm, 6) and (819 nm, 5), predicted NNI values with RMSEP, RRMSE, and REP values of 0.12, 13.4%, and 10.68%, respectively. The validation result of the direct method was better than that of the indirect method. The two new models provided enhanced accuracy and stability in estimating the NNI of summer maize with a simplified and applicable formulation.

## 4 Discussion

### 4.1 Theoretical analysis of plant nitrogen diagnosis based on nitrogen nutrition index

Nitrogen plays a crucial role in the growth and development of crops (Ata-Ul-Karim et al., 2016). As a key component of chlorophyll, nitrogen was essential for photosynthesis, the process by which plants convert light energy into chemical energy. It is also a major constituent of amino acids, the building blocks of proteins, which are vital for cell growth and function. An adequate nitrogen supply promotes vigorous vegetative growth, leading to a larger leaf area, enhanced root development, and overall improved plant health. Conversely, nitrogen deficiency could result in stunted growth, yellowing of leaves (chlorosis), and reduced crop yields. Thus, N treatments had a significant effect on specific parameters (such as PNC and PDM).

The responses of plant DM accumulation and N uptake varied under different N application conditions. Under low N application, both DM accumulation and N uptake were jointly influenced by the plant’s growth potential and the soil’s N supply capacity. As N application increased, both DM accumulation and N uptake also increased (Justes et al., 1994), showing significant differences as shown in **Table 3**. However, under optimal or excessive N application, plant N uptake was primarily determined by soil N availability, independent of the plant’s growth potential. Conversely, under these conditions, DM accumulation was dictated by the plant’s growth potential and was independent of soil N availability (Plénet and Lemaire, 2000). As a result, while plant N concentration (PNC) continued to increase with higher N application, DM accumulation did not significantly increase once N application reached a critical level (Zhao et al., 2017). Based on the behavior of DM accumulation and PNC under varying N conditions, Lemaire and Salette (1984) introduced the concept of N_c_ dilution concentration, which refers to the minimum N concentration required for maximum crop growth. Plénet and Lemaire (2000) further developed the first N_c_ dilution curve for maize, which has since been widely adopted to diagnose the N status of maize globally.

Nitrogen nutrition index was developed based on N_c_ dilution curve (Equation 1). A statistical significance existed between different N treatments, which is due to the difference of plant DM and PNC. When NNI was equal to or higher than 1, plant N status was considered optimal or excessive, and when NNI was lower than 1, plant N status was considered insufficient. The lower plant DM and PNC were from the low N treatments, which can contribute to the low NNI value. Due to the characteristic of the N_c_ curve, even if the plant DM accumulation of maize was not significantly different under excessive N condition, NNI could still recognize a plant’s excessive N status by comparing PNC with plant N_c_ concentration (Ziadi et al., 2008; Ata-Ul-Karim et al., 2017).

### 4.2 Wavelet features for the estimation of nitrogen nutrition index

The Mexican Hat wavelet may have performed better due to its strong localization in both the time and frequency domains, which makes it particularly effective at detecting subtle variations in crop reflectance at certain scales. In contrast, other wavelets like the Haar or Morlet wavelet may have struggled with capturing these variations due to their different frequency responses or poorer localization properties (Cheng et al., 2011). Including this type of comparative analysis in the “Results” section would not only clarify why the Mexican Hat wavelet was superior but also provide valuable insights into the suitability of different wavelets for agricultural spectral analysis (Ngui et al., 2013).

Nitrogen nutrition index was estimated using four regression types—linear, exponential, power, and logarithmic—in this study. The linear models offer simplicity and ease of interpretation but may not capture complex, non-linear relationships as effectively as exponential models could provide valuable context. Conversely, exponential models might better fit certain data patterns, especially when there are diminishing returns in response to increasing nitrogen levels, but they can be more challenging to interpret and apply. In this study, the regression types of power and logarithmic could not be used to fit the relationship between NNI and wavelet feature (**Figures 7C, D**). This is because there was a negative value appearing in the lower scale of the wavelet feature; the fitting result of power and logarithmic types was invalid between NNI and wavelet feature. The *R* ^2^ value is shown as 0 in **Figures 7C, D** under the lower scale conditions. Cheng et al. (2011) considered that more than 2^10^ scale ought to be discarded because the decomposed components at higher scales do now carry meaningful spectral information. The linear and exponential regression types were fit to develop the relationships between NNI and wavelet feature (**Figures 7A, B**). The *R* ^2^ value of wavelet power (745 nm, 7) deduced from the linear regression was slightly better than that of wavelet power (784 nm, 7) based on exponential regression. The wavelet feature (745 nm, 7) was close to the leading red edge position. Many studies reported that the red edge was very sensitive to the change of PDM and PNC, which can reflect plant N stress (Yao et al., 2010; Pellissier et al., 2015; Li et al., 2017). The feature (745 nm, 7) carried information of reflectance spectra across the red edge and near-infrared region (715 to 775 nm) that is mainly controlled by crop N stress and biophysical parameters (PDM) centered at 740 nm (Thenkabail et al., 2004; Chan and Paelinckx, 2008).

High-scale wavelet features are particularly adept at capturing large-scale patterns in the spectral reflectance data, which are often influenced by the overall structure of the crop, such as leaf area, canopy density, and plant height. These structural characteristics can significantly impact how light interacts with the crop, affecting the absorption and reflection of different wavelengths. Cheng et al. (2011) explained that low-scale components are suitable for capturing the characteristics of narrow absorption features, while high-scale components are better suited for defining the overall spectral shape of the canopy spectra. Since NNI reflects the canopy structure throughout the crop growth process, the higher-scale wavelet features in this study were found to be particularly effective in establishing a relationship with NNI. These high-scale features can capture the impact of crop structure on the amplitude of reflectance spectra and partially reduce the influence of the biochemical absorption characteristics (Li, 2016). Consequently, using high-scale wavelet features helps maintain more stable NNI estimations.

In this indirect method, the wavelet features (819 nm, 5) for PDM and (581 nm, 6) for PNC were determined to develop the relationships between wavelet feature and NNI. The power and logarithmic regression types could not be used to fit the relationships between PDM, PNC, and wavelet feature (**Figures 9C, D**, **8C, D**). The wavelet feature (819 nm, 5) was located at the near-infrared region (814 to 824 nm) of the reflectance spectra and was close to the sensitive region of the wavelet feature based on NNI. There was a significantly positive relationship between leaf area index and PDM during the V6 to V12 stages of summer maize. The amplitude of the reflectance spectra was also affected by PDM, which made the sensitive wavelet feature to PDM influenced by other biophysical parameters (leaf area index). The sensitive wavelet feature (581 nm, 6) to PNC was located at the green light region (567 to 587 nm) of the reflectance spectra and was close to the strong reflected peak (550 nm) of chlorophyll, which has been identified as a sensitive wavelength for N estimation in previous studies (Huang et al., 2004). During the vegetative growth stage of crops, plants invest a higher proportion of photosynthate in the structural compartment (low N concentration), and more absorbed N was transported into the upper leaves due to plant light distribution (Lemaire et al., 2008). Therefore, PNC decreased gradually with plant growth. Due to the capture capacity of the high scale of wavelet feature to the information of reflectance amplitude, the estimation of PNC was more suitable using a higher scale based on continuous wavelet transform method.

### 4.3 Comparison with the traditional method and existing wavelet feature

The change of spectral reflectance from visible to near-infrared region could represent the change of plant N status. In the visible region, changes in reflectance are often linked to chlorophyll content, which directly correlates with nitrogen status. A decrease in nitrogen can lead to chlorosis, reducing chlorophyll and thereby increasing reflectance in the green and red bands. In the near-infrared region, reflectance is largely influenced by the internal structure of plant leaves, including cell density and water content. N deficiency can alter these structural properties, leading to changes in near-infrared reflectance.

In this study, the three existing spectral indices were used to estimate NNI of summer maize using the calibration data set (**Table 2**). The result confirmed that these indices could represent the change of NNI, but the performance was not unsatisfactory (**Supplementary Figure S4**). The strongest relationship was found between MTCI and NNI. The *R* ^2^ value was 0.61, which was lower than 0.7. The performance of the developed wavelet feature (*R* ^2^ value higher than 0.8) was better than the existing spectral indices (**Figure 7**). The first reason was that the traditional spectral indices were not specifically designed to estimate NNI; they were originally developed to assess parameters like PNC or PDM across different crops (Mistele and Schmidhalter, 2008; Chen, 2015; Liu et al., 2018). The second reason was that these traditional indices typically rely on two or three independent spectral bands to evaluate crop growth status. This approach was highly susceptible to external environmental factors and cultivar characteristics, leading to inconsistent and unstable predictive performance of the spectral indices. The CWA method was particularly effective because it could isolate and capture relevant features at different scales, allowing it to account for variations in crop structure and minimize the influence of noise or irrelevant spectral information. This led to more consistent and reliable NNI predictions across varying conditions. The contained spectral information of the single spectral band was very limited, and the reflectance of the neighboring spectral bands might be different with the determined spectral band, which led to substantial decreases in the predictive performance to the same objective (Cheng et al., 2014a).

In the indirect method, PDM and PNC were estimated using wavelet features based on continuous wavelet transform, respectively. The wavelet feature (581 nm, 6) was the most sensitive feature to PNC. The wavelet features based on the green light region have been reported as a sensitive feature to estimate N or chlorophyll content by many studies (Liao et al., 2013; Wang et al., 2016, 2017). All *R* ^2^ values between wavelet features and PDM were lower than 0.7 (**Figures 9A, B**), and their performance was also weaker than the two other indices estimated (NNI and PNC), which indicated that the better wavelet feature to estimate PDM might be located at the longer wavelengths (shortwave infrared region) of the reflectance spectra. The decomposition scale (5) of this study was similar with that (4 or 5) determined by Cheng et al. (2014b). The correlation between dry matter and wavelet feature was better for scale 5 than for a higher scale. The most sensitive wavelet feature (819 nm, 4) that integrated reflectance information from the spectral segment (814–824 nm) characterized the change of dry matter in spectral shape more efficiently than the traditional spectral indices (**Supplementary Figure S5**). This could eliminate the effect of canopy structure on spectral segment and therefore was highly promising for canopy-level applications.

Through the calibration and validation of the relationships between NNI and wavelet feature using field data set, each of the direct and indirect methods could assess the NNI of summer maize, but the performance of the direct method was better than that of the indirect method. External environmental factors such as variations in weather conditions, soil heterogeneity, and unexpected pest or disease outbreaks could introduce variability that impacts the predictive accuracy of the developed models in this study. Additionally, errors during data collection, such as inaccuracies in sensor readings or sampling inconsistencies, could further contribute to discrepancies between the predicted and observed outcomes. These sources of error could lead to overestimation or underestimation of certain variables, thereby affecting the model’s overall reliability. These factors would not only highlight the limitations of the current model but also suggest areas for improvement, such as refining data collection methods or incorporating additional environmental variables into the model. Addressing these potential errors is crucial to enhance the model’s predictive ability and ensure its robustness in different scenarios.

The combination structure and calculation method for the newly developed wavelet feature (745 nm, 7) were simpler than those of the two wavelet features (581 nm, 6) and (819 nm, 5), which could reduce the risk of NNI prediction. This new wavelet feature will be useful to design an exclusive index to diagnose the plant N status of summer maize. However, due to the limitation of the experiment data and spectral band, future work is required to test the adaptation and reliability of the newly developed wavelet feature under diverse external environments.

## 5 Conclusion

This study confirmed the effectiveness of using wavelet features to predict the nitrogen nutrition index (NNI) in summer maize through the continuous wavelet analysis (CWA) method, with the Mexican Hat wavelet identified as the most suitable mother wavelet. In the direct method, the most sensitive wavelet feature (745 nm, scale 7) was identified to assess NNI, and the linear regression model established was NNI = 0.14 WF (745 nm, 7) + 0.3. In the indirect method, wavelet features (819 nm, scale 5) to predict PDM and (581 nm, scale 6) for PNC were used to construct the calculation model for NNI. The two methods of NNI estimation were compared by using independent data sets. The result indicated that the performances of the direct and indirect estimation methods were accurate and stable. Compared with the established spectral indices, Mexican Hat is shown to have a more effective capacity in collecting meaningful spectral information that relates to NNI, PDM, and PNC. By decomposing the reflectance spectra into various scales, the high scale features could capture the information of the reflectance amplitude based on the shape of the spectral curve, and the low scale features could capture the absorption characteristics of the objective index (PNC and PDM). The result of this study revealed that the wavelet feature of NNI successfully differentiated the different plant N status of summer maize. The CWA method was extended to the field of plant N diagnosis and enlarged its application range. Further research could focus on optimizing the wavelet analysis method by exploring different wavelet functions or scales to enhance its predictive accuracy. Moreover, validating the method across a broader range of crops and environmental conditions would help establish its generalizability and practical applicability in diverse agricultural settings.

## Data Availability

The original contributions presented in the study are included in the article/**Supplementary Material**. Further inquiries can be directed to the corresponding author.
