# Supplementary material for: Advancing nitrogen nutrition index estimation in summer maize using continuous wavelet transform
Source: Front Plant Sci. 2024 Nov 11;15:1478162. doi: 10.3389/fpls.2024.1478162 (PMC11586213; doi:10.3389/fpls.2024.1478162)
Supplement: Supplementary file 1 [file DataSheet1.docx]

***Supplementary Material***

Fig. S1 the relationships between NNI and wavelet feature at (745 nm, 7) of the linear regression (A) and (784nm,7) of the exponential regression (B) established with the calibration data set.

Fig. S2 the relationships between plant dry matter and wavelet feature at (819nm, 5) of the linear regression (A) and (782nm,3) of the exponential regression (B) established with the calibration data set.

Fig. S3 the relationships between plant nitrogen concentration and wavelet feature at (581nm, 6) of the linear regression (A) and (573nm,6) of the exponential regression (B) established with the calibration data set.

Fig. S4 the relationships between nitrogen nutrition index and spectral indices calculated from calibration data set.

Fig. S5 Wavelength location of wavelet features within each region for NNI , PDM and PNC. The center (dashed vertical line) and the width of a mother wavelet (Mexican Hat) curve indicate the wavelength location and scale of each wavelet feature. The two numbers beside each vertical line are wavelengths of start and end points of the wavelet feature.
